# Preparation of Hierarchically Porous Graphitic Carbon Spheres and Their Applications in Supercapacitors and Dye Adsorption

**DOI:** 10.3390/nano8080625

**Published:** 2018-08-17

**Authors:** Saisai Li, Faliang Li, Junkai Wang, Liang Tian, Haijun Zhang, Shaowei Zhang

**Affiliations:** 1 The State Key Laboratory of Refractories and Metallurgy, Wuhan University of Science and Technology, Wuhan 430081, China; lisaisai281024@163.com (S.L.); lfliang@wust.edu.cn (F.L.); jkwang@hpu.edu.cn (J.W.); 13657244966@163.com (L.T.); 2 College of Engineering, Mathematics and Physical Sciences, University of Exeter, Exeter EX4 4QF, UK

**Keywords:** graphitic carbon spheres, catalytic graphitization, hierarchical pores, supercapacitors, dye adsorption

## Abstract

Hierarchical micro-/mesoporous graphitic carbon spheres (HGCS) with a uniform diameter of ~0.35 μm were synthesized by Fe-catalyzed graphitization of amorphous carbon spheres resultant from hydrothermal carbonization. The HGCS resultant from 3 h at 900 °C with 1.0 wt % Fe catalyst had a high graphitization degree and surface area as high as 564 m^2^/g. They also exhibited high specific capacitance of 140 F/g at 0.2 A/g and good electrochemical stability with 94% capacitance retention after consecutive 2500 cycles. The graphitization degree of the HGCS contributed to 60% of their specific capacitance, and their specific capacitance per unit surface area was as high as 0.2 F/m^2^, which was much higher than in the most cases of porous amorphous carbon materials reported before. In addition, the HGCS showed a high adsorption capacity of 182.8 mg/g for methylene blue (MB), which was 12 times as high as that in the case of carbon spheres before graphitization.

## 1. Introduction

As an important electrochemical energy storage device, supercapacitors are attracting more and more attention [1,2]. Their electrode materials play a significant role in their performance [3,4,5]. Porous carbon materials are one of the most commonly used supercapacitor electrode materials because of their large surface area (S_BET_ > 1000 m^2^/g) which can improve the accumulation of ions at the electrode/electrolyte interface via forming electrical double layers [6]. Generally, the specific capacitance increases with increasing the surface area. Pang et al. prepared hierarchical nitrogen-doped carbon spheres with specific surface area of 1939 m^2^/g, which showed a superior specific capacitance as high as 165 F/g at 1 A/g [1]. Zhang et al. synthesized activated porous carbon using KOH as a deoxidant and activation agent. The material had a specific surface area of 1672 m^2^/g and exhibited a maximum specific capacitance of 226 F/g at 1 A/g [7]. Despite these promising results, the strategy to increase the capacitance by increasing electrode material’s specific surface area is approaching its limit. Therefore, alternative strategies need to be developed to further improve specific capacitance of porous carbon materials.

Consequently, an alternative strategy via enhancing graphitization degree of porous carbon materials was proposed, and several types of hierarchical graphitic carbons with well-controlled crystalline structure, large surface area, high porosity, high electric conductivity, and good chemical and electrochemical stability were developed [8,9,10,11,12,13,14]. For instance, Xie et al. synthesized porous graphitic carbon materials with enhanced electrochemical performances by improving the graphitization degree using Fe and Ni catalysts [15]. Estevez et al. prepared hierarchically porous graphitic carbon using colloidal silica and ice as templates, along with graphitization and physical activation at 1000 and 900 °C, respectively. The material exhibited stable performance when it was used to build a supercapacitor electrode [10]. Nevertheless, the preparation processes developed for hierarchical graphitic porous carbon materials were very complex, involving multiple steps and requiring preformed templates, such as porous polymers and silica monoliths [16,17,18].

To address the issue, in this work, hierarchical graphitic carbon spheres (HGCS) were prepared via combined hydrothermal carbonization and catalytic graphitization using glucose and Fe(NO_3_)_3_·9H_2_O as the carbon source and catalytic precursor, respectively. Their multifunctional application potentials were demonstrated by examining their electrochemical performance and adsorption capacity for toxic dye.

## 2. Experimental Section

### 2.1. Preparation of HGCS

The main starting materials used included glucose (C_6_H_12_O_6_·H_2_O, AR; Bodi chem. Co., Ltd., Tianjin, China), commercial ferric nitrate (Fe(NO_3_)_3_·9H_2_O, 99.0%, Lia Chemical Co., Ltd., Wuhan, China), sodium polyacrylate (PAANa, (C_3_H_3_O_2_Na)n, AR; Damao chem. Co., Ltd., Tianjin, China), methylene blue trihydrate (MB, C_16_H_18_ClN_3_S·3H_2_O, Sinopharm chem. Co., Ltd., Shanghai, China), and potassium hydroxide (KOH, AR; Sinopharm Co., Ltd., Shanghai, China).

The preparation process of HGCS typically involved the following two steps: (1) low temperature hydrothermal carbonization to prepare carbon spheres and (2) high temperature catalytic graphitization of presynthesized carbon nanospheres to obtain HGCS. In the first step, an aqueous solution of glucose (0.7 mol/L) was combined with PAANa (1.0 wt % with respect to carbon) forming a transparent solution. Since PAA anions can reduce the surface energy of carbon spheres [19], PAANa was chosen as the dispersant in the present case. 70 mL of the resultant solution (70 mL) were introduced into a stainless-steel autoclave and subjected to hydrothermal carbonization at 180 °C/12 h. The reaction product was washed repeatedly with ethanol and deionized water, collected via filtration and dried at 80 °C/12 h. The carbon spheres so made had an average size of 0.48 μm and a specific surface area of 12 m^2^/g (Figures S1 and S2). To prepare HGCS, various amounts of Fe(NO_3_)_3_·9H_2_O (0.5–2.0 wt % Fe with respect to carbon spheres) were dissolved in 50 mL alcohol under 25 °C, forming a homogeneous solution to which the presynthesized carbon spheres were carefully added to form a uniform gel. The gel was dried at 80 °C/12 h, and then fired at a heating rate of 5 °C/min to a given temperature (800–1100 °C) and held for 1–5 h in an alumina tube furnace protected by flowing Ar (99.999 vol % pure). The resultant HGCS were subject to characterization directly without any further purification.

### 2.2. Sample Characterization

The crystalline phases in samples were identified by powder X-ray diffraction (XRD) analysis using a Philips X’Pert PRO diffractometer (Xpertpro, PHILIPS, Hillsboro, The Netherlands). The morphologies of samples were observed by means of a field-emission scanning electron microscope (FE-SEM; Nova400NanoSEM, 15 kV, Philips, Amsterdam, The Netherlands) and transmission electron microscope (TEM; JEM-2100UHRSTEM, 200 kV, JEOL, Akishima, Tokyo, Japan) equipped with an energy dispersive spectrometer (EDS, Penta FET X-3 Si (Li)). Raman spectra were recorded using a Horiba Jobin-Yvon Labram-HR800 Raman spectrometer (Raman, Paris, France); a 532 nm diode laser was used with an integration time of 30 s, a spectral resolution of 1 nm, and an approximate power level of 2 mW. To calculate the mean diameter and determine the size distribution, at least 200 carbonaceous spheres were examined in each case. Nitrogen adsorption–desorption isotherms were determined (Autosorb-1-MP/LP, Quantachrome, FL, USA) to calculate the specific surface area and pore size distribution. The specific surface area was calculated according to the Brunauer Emmett Teller (BET) model and the pore size distribution was determined using the Barret–Joner–Halenda (BJH) method. The MB adsorption was evaluated using an ultraviolet-visible spectrophotometer (UV-vis, UV-2550, Shimadzu Corporation, Kyoto, Japan).

### 2.3. Electrochemical Measurement

Electrochemical behavior of as-prepared HGCS was investigated in a three electrode configuration at 25 °C using Hg/HgO, Pt foil, and 6M KOH aqueous solution respectively as the reference electrode, the counter electrode, and the electrolyte. The working electrode was prepared by coating carbon spheres and HGCS with conducting nickel foam, and then used directly in the circuit. Cyclic voltammetry (CV) and galvanostatic charge/discharge (GCD) curves were used to evaluate electrochemical performances of samples. The former was determined between −1.0 and 0 V potential window at scan rates of 5, 10, 20, 50, 100, and 200 mV/s, and the latter determined between −1.0 and 0 V at current densities of 0.1, 0.2, 0.5, 1, 2, 5, and 10 A/g.

Based on the CV and GCD curves, the specific capacitance values were calculated by the following equations:

(1) $$C=\frac{\int IdV}{vmV}$$

(2) $$C=\frac{I\Delta t}{mV}$$

where *C* (F/g) is the specific capacitance, *I* (A) the instant current shown in the CV curve, *m* (g) the sample mass in the working electrode, *v* (V/s) the scan rate, Δ*t* (s) the discharge time, and *V* (V) the voltage upon discharging.

The cycling stability was evaluated based on the capacitance retention after 2500 consecutive galvanostatical charge and discharge cycles at 5 A/g.

### 2.4. Adsorption Test

The adsorption tests were carried out using MB as an adsorbate. Typically, 20 mg of as-prepared samples were added into an MB solution with different concentrations (0–50 mg/L), and stirred at 25 °C for 30 min. After centrifugation, the concentration of supernatant solution was measured using a UV-visible spectrophotometer. The adsorption kinetics was also investigated and adsorption isotherms were determined following the procedures reported in our earlier publication [20].

## 3. Results and Discussion

### 3.1. Effect of Heating Temperature on Graphitization

**Figure 1:**
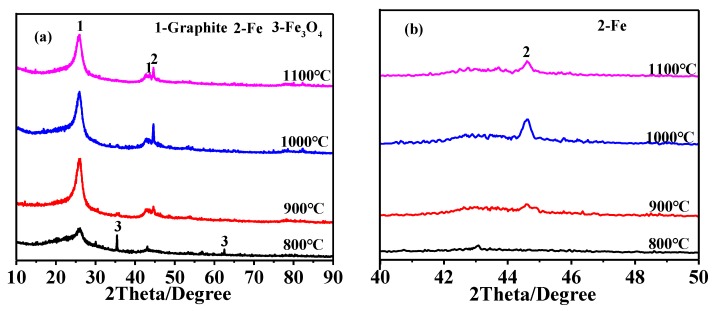
(**a**) XRD patterns of HGCS resulted from 3 h firing of presynthesized carbon spheres with 1.0 wt % Fe at different temperatures and (**b**) magnification of the XRD patterns within 40–50° (ICDD: 01-075-1621 (Graphite), 01-089-7194 (Fe), and 01-089-0688 (Fe_3_O_4_)).

The carbon spheres presynthesized via hydrothermal carbonization (Figure S1) were thermally graphitized at different temperatures for 3 h, with and without the Fe catalyst. In the catalyst-free case, two broad peaks (centered at about 2θ = 26 and 43° (2θ)) corresponding to low crystallinity graphite appeared in the XRD pattern, and they almost did not change with increasing heating temperature from 800 to 1100 °C (Figure S3), indicating little effect of heating temperature on the graphitization of carbon spheres. In contrast, in the case of using 1.0 wt % Fe catalyst, the two diffraction peaks became much sharper and higher, upon increasing the heating temperature from 800 to 900 °C (Figure 1a), indicating the great effect of heating temperature (along with the catalyst) on the graphitization of carbon spheres (HGCS). From these XRD results, it can be concluded that the carbon spheres were changed from amorphous carbon to graphitic carbon upon heat treatment with the Fe catalyst, via a dissolution and precipitation mechanism, i.e., the initial amorphous carbon dissolved in the Fe catalyst and subsequently precipitated from the saturated catalyst as graphitic carbon [21]. Nevertheless, further increasing the temperature to above 900 °C did not result in any obvious changes in the shape and height of the two peaks, i.e., almost no further improvement in the graphitization degree above 900 °C. In addition to graphitic carbon, Fe_3_O_4_ was detected in the CS fired at 800 °C, but it was replaced by Fe at a higher heating temperature (>800 °C) (Figure 1b), suggesting that Fe(NO_3_)_3_·9H_2_O had initially decomposed at a low temperature (800 °C) to Fe_3_O_4_ which was further reduced at a higher temperature to Fe catalyst [22,23].

**Figure 2:**
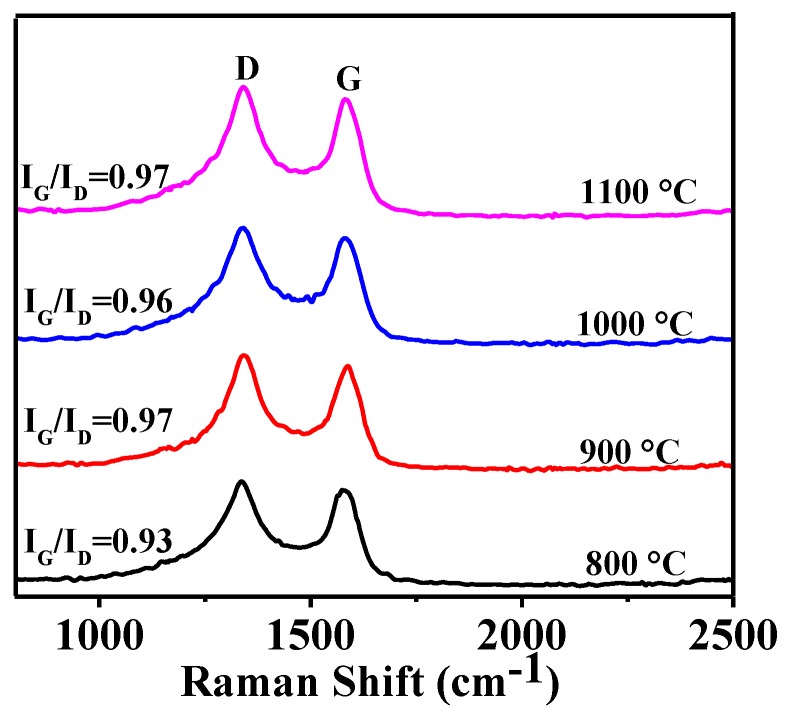
Raman spectra of HGCS whose XRD patterns are given in Figure 1.

Raman spectra of HGCS resulted from graphitization of carbon spheres revealed D and G bands at around 1350 and 1590 cm^−1^, respectively (Figure 2). The former arose from the disorder-induced defects of graphitic carbon while the latter from the sp^2^-hybridized carbon in HGCS, and the intensity ratio of the former (I_G_) to the latter (I_D_) (I_G_/I_D_) indicated the graphitization degree of HGCS [24]. According to Figure 2, the I_G_/I_D_ values of HGCS resultant from 3 h firing at 800, 900, 1000, and 1100 °C were determined as 0.93, 0.97, 0.96, and 0.97, respectively, indicating that the graphitization degree of the samples increased initially with increasing the temperature from 800 to 900 °C, but almost did not change upon further increasing the temperature to above 900 °C, which, along with the XRD results (Figure 1) described and discussed above, suggested that the optimal graphitization temperature for HGCS preparation was 900 °C in this work.

### 3.2. Effect of Catalyst Content on Graphitization

**Figure 3:**
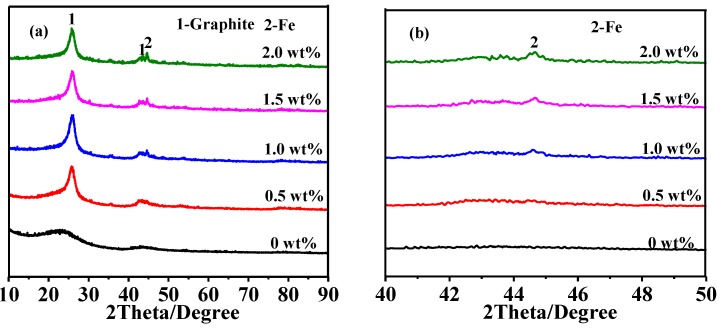
(**a**) XRD patterns of HGCS resulted from 3 h firing at 900 °C with various amounts of Fe catalyst and (**b**) magnification of the XRD patterns within 40–50° (ICDD: 01-075-1621 (Graphite) and 01-089-7194 (Fe)).

**Figure 4:**
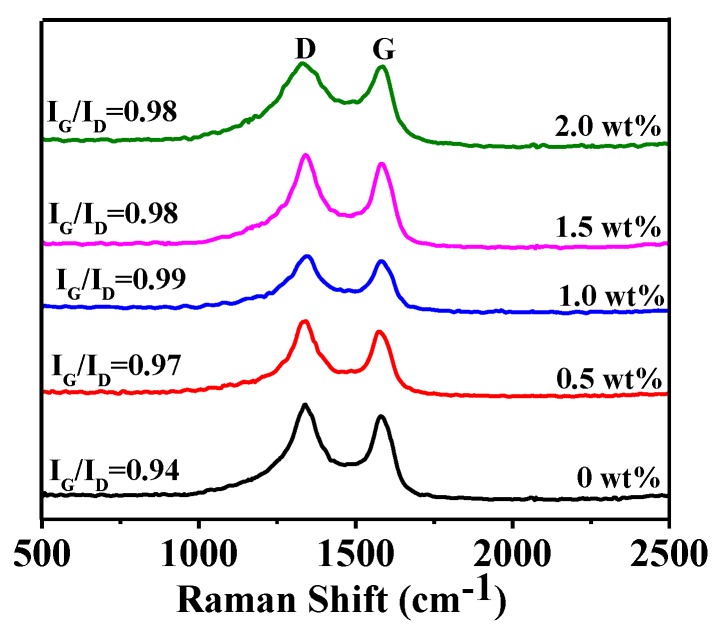
Raman spectra of HGCS whose XRD patterns are given in Figure 3.

Figure 3 shows XRD patterns of HGCS resulted from 3 h firing at 900 °C with various amounts of Fe catalyst. It can be seen that the peak heights of graphite increased with increasing the Fe catalyst from 0 to 1.0 wt %, indicating the gradually enhanced graphitization degree of HGCS. However, they adversely decreased upon further increasing the Fe catalyst to 1.5–2.0 wt %, which might be caused by the aggregation and deactivation of the Fe nanoparticles due to their excessive amount [25]. Raman spectra of the samples (Figure 4) further revealed that the maximal I_G_/I_D_ ratio (0.99) was reached upon using 1.0 wt % Fe catalyst. These results (Figure 3 and Figure 4) indicated that the optimal amount of Fe catalyst for preparation of HGCS was 1.0 wt %.

### 3.3. Effect of Soaking Time on Graphitization

**Figure 5:**
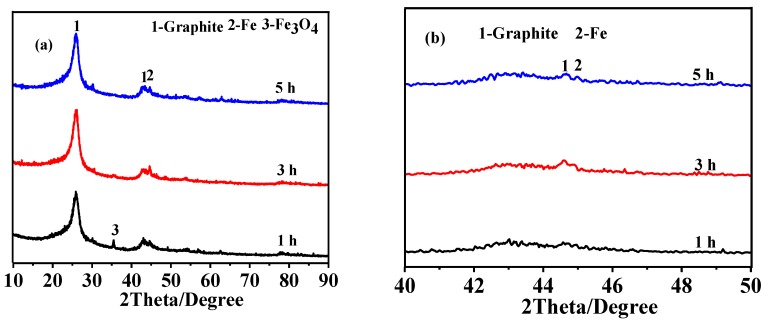
(**a**) XRD patterns of HGCS resulted from heat treatment of presynthesized carbon spheres with 1.0 wt % Fe at 900 °C for different times and (**b**) magnification of the XRD patterns within 40–50° (ICDD: 01-075-1621 (Graphite), 01-089-7194 (Fe), and 01-089-0688 (Fe_3_O_4_)).

**Figure 6:**
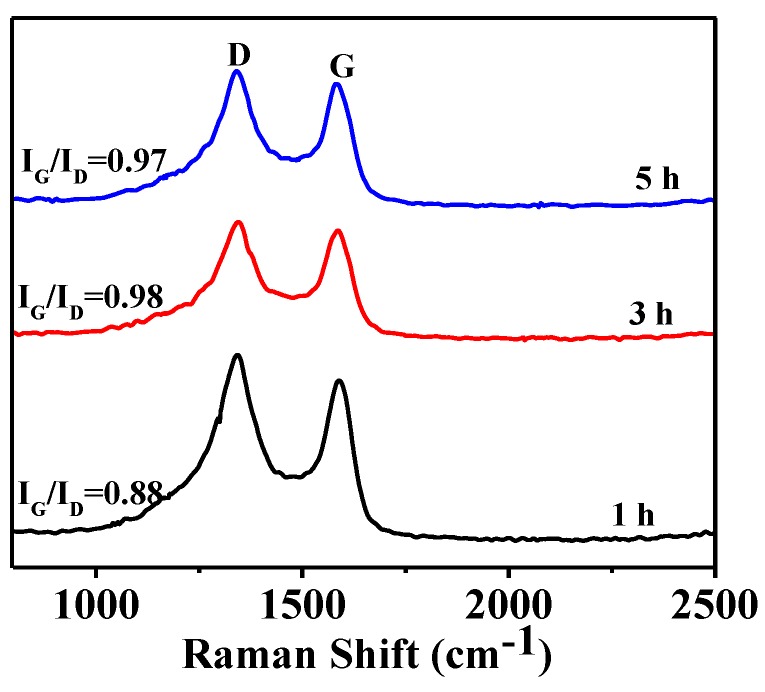
Raman spectra of HGCS whose XRD patterns are given in Figure 5.

Given in Figure 5 are XRD patterns of HGCS fired with 1.0 wt % Fe catalyst at 900 °C for different time periods, showing that graphite was the main product in all the samples. After 1 h at 900 °C, some Fe_3_O_4_ still remained in the fired sample (Figure 5a). It was reduced to Fe with extending the time further to 3 and 5 h (Figure 5b), associated with which the peak heights of graphite, i.e., graphitization degree, also increased. The corresponding Raman spectra of HGCS samples (Figure 6) also indicated that the I_G_/I_D_ ratio increased from 0.88 to 0.98 and then decreased to 0.97 with extending the soaking time from 1 to 3 h, and to 5 h. The above results suggested that 3 h was the optimal soaking time in the present case.

Based on the results shown in Figure 1, Figure 2, Figure 3, Figure 4, Figure 5 and Figure 6 and discussed above, it could be considered that the optimal graphitization condition in the present work was: 3 h firing at 900 °C with 1.0 wt % Fe catalyst.

### 3.4. Microstructural Characterization of As-Prepared HGCS

**Figure 7:**
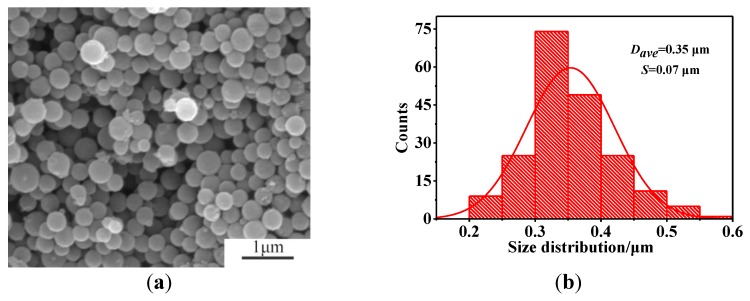
(**a**) SEM image and (**b**) particle size distribution of HGCS prepared at 900 °C for 3 h with 1.0 wt % Fe catalyst.

**Figure 8:**
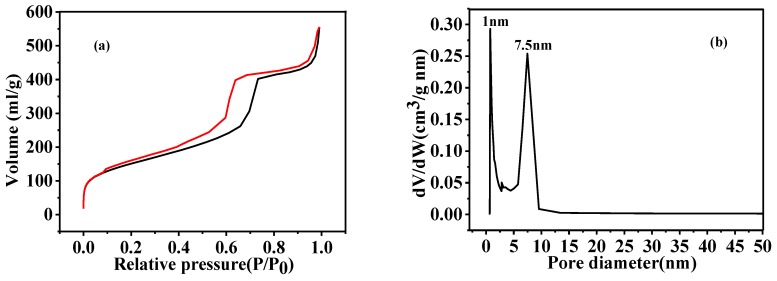
N_2_ adsorption–desorptionadsorption–desorption isotherm (**a**) and pore size distribution curves (**b**) of HGCS prepared at 900 °C for 3 h with 1.0 wt % Fe catalyst.

The SEM image presented in Figure 7 shows the HGCS prepared at the optimal conditions (3 h firing at 900 °C with 1.0 wt % Fe catalyst), revealing the formation of uniform spheres with an average diameter of about 0.35 μm, which was slightly smaller than that of the presynthesized carbon spheres (0.48 μm). Furthermore, their specific surface area and pore size distribution were determined by nitrogen adsorption–desorption tests. As seen from Figure 8a, the N_2_ sorption isotherms showed that both micropores (the sharply increased adsorption at relative pressures close to 0) and mesopores (the adsorption in the relative pressure range of 0.1–0.8) were present in the HGCS [26,27]. Given in Figure 8 are pore size distribution curves determined from desorption isotherms using the BJH method, showing that the sizes of micropores were centered at about 1 nm and those of the mesopores were mainly around 7.5 nm, which further demonstrated the formation of a hierarchical micro-/mesoporous structure in the present HGCS. Moreover, the specific surface area of the HGCS was determined from a multipoint BET plot as about 564 m^2^/g. Table S1 compares graphitic carbon materials prepared previously by the template method and the present catalytic method, indicating that the HGCS prepared in this work possessed reasonably large surface area. Although the graphitic carbon materials prepared by the template method had relatively larger specific surface area, their preparation processes were complex and we had to use strong acids or alkalis to remove the template. They also hardly exhibited regular shape and homogeneous size distribution.

**Figure 9:**
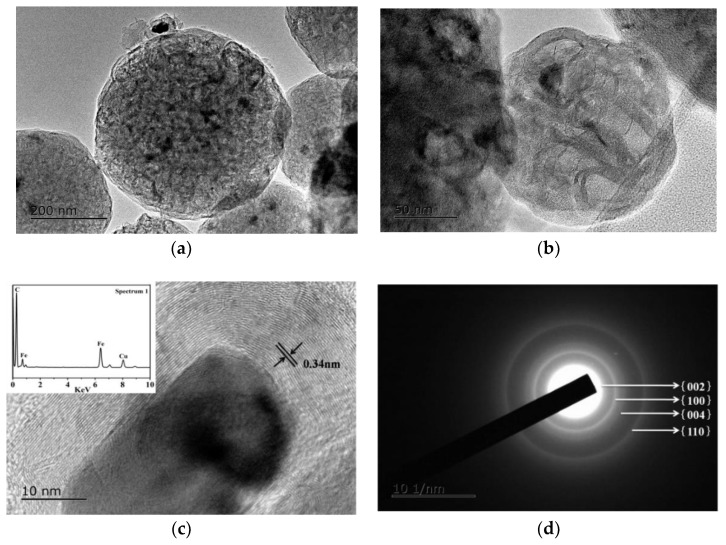
TEM (**a**), HRTEM images (**b**), selected-are electron diffraction (SAED) (**c**), and energy dispersive spectrometer (EDS) (**d**) of HGCS prepared at 900 °C for 3 h with 1.0 wt % Fe catalyst.

In order to better understand the morphology and structure of the HGCS sample, TEM analysis was carried out. As shown in Figure 9a, graphitic carbon spheres in the sample were about 0.4 μm in size. Also, numerous bright contrast spots of several nanometers were observed, demonstrating the existence of micropores and mesopores in the HGCS (Figure 9b). These results can be correlated with the BET results shown in Figure 8. Moreover, many black contrast spots of ~20 nm were present in the HGCS. EDS results (Figure 9c inset) indicated that they were Fe nanoparticles. The HRTEM image in Figure 9c demonstrated the high degree of crystallinity of HGCS. The well-resolved lattice fringes surrounding the Fe nanoparticle showed an interplanar spacing of 0.34 nm, which corresponded to the d-spacing of {002} plane of graphite. Furthermore, the selected-area electron diffraction (SAED) pattern (Figure 9d) showed well-defined diffraction rings; the centric one corresponded to the {002} plane, and the other ones corresponded to {100}, {004}, and {110} planes of graphite, indicating the polycrystal nature of the HGCS.

**Figure 10:**
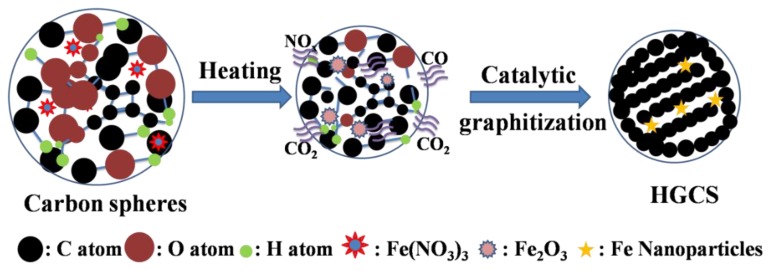
Formation mechanism of HGCS in the case of using Fe catalyst.

As stated above, TEM (Figure 9) reveals much more micropore and mesopores in the HGCS than in the presynthesized carbon spheres (Figure S4), which is corresponding with the BET results. The HGCS showed sharply increased specific surface area (from 12 to 564 m^2^/g), which could be explained as follows. (1) After catalytic graphitization, the average size of the HGCS was decreased (from 0.48 to 0.35 μm) (Figure 7 and Figure S1), and correspondingly about 50% weight was lost with respect to the presynthesized carbon spheres, indicating that a significant number of original oxygen containing groups were lost during the graphitization process, creating lots of new pores in the HGCS [20,28]; (2) the Fe(NO_3_)_3_ 9H_2_O precursor not only acted as a catalyst, but also as an oxidizing agent during the graphitization process. It reacted with carbon spheres, releasing some gaseous phases such as NO_x_, CO, and CO_2_, which was beneficial to the physical activation of micro/meso pores formation [10]; and (3) comparison of Figure S4 and Figure 9a revealed that, after catalyzed graphitization, the disordered carbon atoms in the original presynthesized amorphous carbon spheres were rearranged into regular graphite layers, which believed to have left some tiny gaps between the amorphous carbon layer and the regular graphite layer, and thus increased the specific surface area of the HGCS. Based on above results and discussion, a possible pore formation mechanism in the present work is proposed and schematically illustrated in Figure 10.

### 3.5. Electrochemical Performance of as Prepared HGCS

**Figure 11:**
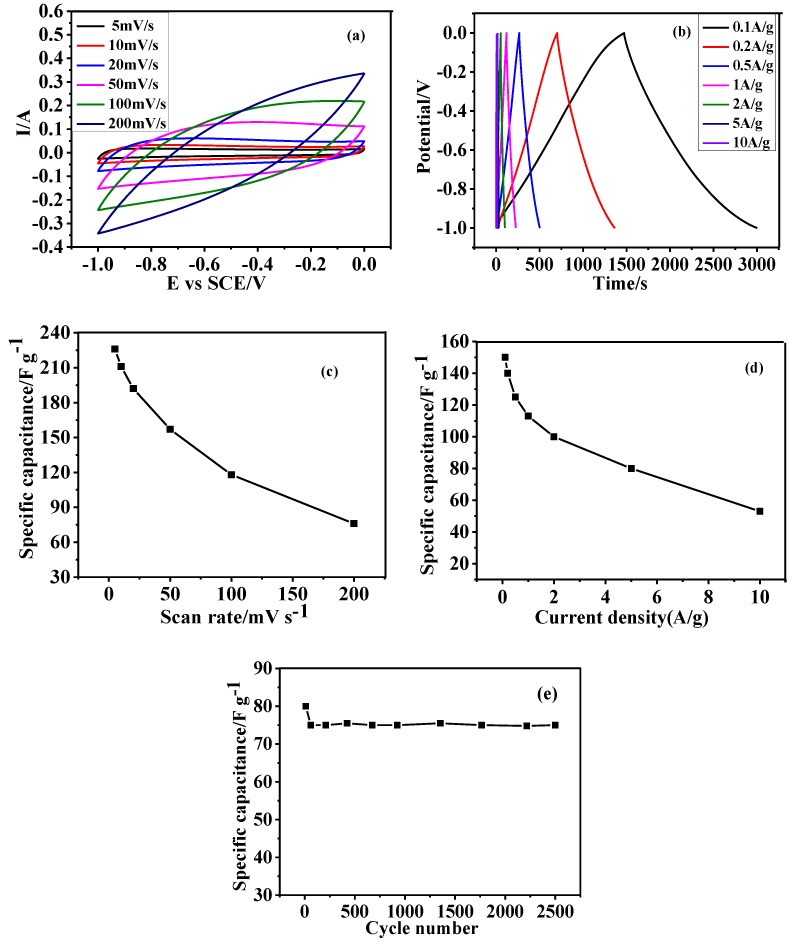
CV curves (**a**), galvanostatic charge/discharge curves (**b**), specific capacitance versus scan rate (**c**), specific capacitance versus current density (**d**), and cycling performance at current density of 5 A/g of as-prepared HGCS (**e**).

The capacitive properties of the starting carbon spheres (180 °C/12 h) and as-prepared HGCS (900 °C, 3 h, and 1.0 wt % Fe) were respectively studied by cyclic voltammetry (CV) at various scan rates from 5 to 200 mV·s^−1^ in the potential window from −1.0 to 0 V and galvanostatic charge-discharge (GCD) measurements at various current densities from 0.1 to 10 A/g. The distorted quasi-rectangular CV and asymmetric triangular GCD curves in the case of presynthesized carbon spheres (Figure S5a,b and Figure 11a,b) were mainly due to the oxygen-containing functional groups on the carbon spheres, which might change the potential of positive electrode during the charge/discharge cycles of supercapacitors [29,30]. As for the sharply decreased specific capacitance (all less than 75 F/g (Figure S5c,d)), it can be explained as follows: (1) The electrostatic repulsion and steric hindrance inhibited the transportation contacting of OH^−^ and the electrodes; and (2) the presynthesized carbon spheres were solid and their specific surface area was low (Figures S2 and S4), which affected negatively the storage of electrolytes in the electrodes.

On the other hand, in the case of the HGCS, the quasi-rectangular and symmetric CV curves (Figure 11a,b) without any redox peaks indicated that their capacitive properties were determined by the electrical double-layer capacitors. As shown in Figure 11c, the specific capacitance of the HGCS decreased from 226 to 76 F/g with increasing the scan rate from 5 to 200 mV/s, which were 2–3 times higher than in the case of presynthesized carbon spheres. Moreover, the specific capacitance at 5 mV/s was also slightly higher than that of activated carbon reported previously (200 F/g at 5 mV/s, BET = 3263 m^2^/g) [31], although the specific surface area in the case of the former was far lower than in the case of the latter. The higher specific capacitance of as-prepared HGCS was attributed to their higher graphitization degree and better conductivity, implying that the graphitization degree of a carbon material played an important role in improving its specific capacitive performance. Moreover, the specific capacitance values at different current densities were also calculated (150 F/g at 0.1 A/g, 140 F/g at 0.2 A/g, 125 F/g at 0.5 A/g, 113 F/g at 1 A/g, 100 F/g at 2 A/g, 80 F/g at 5 A/g, and 53 F/g at 10 A/g). The value at 0.2 A/g was higher than that of the porous graphitic carbon material reported previously (120 F/g at 0.2 A/g, BET = 329 m^2^/g) [15], even though the graphitization degree was higher in the case of the latter. This result suggested that the hierarchically porous structure and high specific surface area of the HGCS, which led to the intimate contact between the electrolyte and the electrode, were also important to their specific capacitance performance. Based on above results, it can be concluded that graphitization degree, as well as hierarchically porous structure and specific surface area, is of fundamental importance to the electrical capacitive performances of carbon materials. In addition, it can be concluded that after heat treatment, the specific capacitance of the resultant HGCS became higher, although the improvement was still limited, due to their still relatively low specific surface area.

To evaluate the effect of graphitization degree of HGCS on their electrochemical performance, the specific capacitance values of amorphous mesoporous carbon spheres and as-prepared HGCS with similar specific surface area were compared (S_BET_ = 666 m^2^/g [32] for the former, and S_BET_ = 564 m^2^/g for the latter). The former showed a low specific capacitance of 59 F/g at 0.2 A/g which was similar to that of the presynthesized carbon spheres (prepared at 180 °C for 12 h). Since the specific capacitance of the HGCS at 0.2 A/g was 140 F/g, it can be reasonably estimated that the graphitization degree contributed to 60% of their specific capacitance. Moreover, the surface area normalized specific capacitance (Cs, F/m^2^) was calculated according to the equation of Cs = C/S_BET_, where C and S_BET_ are the specific capacitance and the BET surface area, respectively. As demonstrated in Table S2, Cs of the HGCS was as high as 0.2 F/m^2^, which was much higher than in the cases of most documented porous amorphous carbon materials, such as activated porous carbon (0.13 F/m^2^) [7] and hierarchically porous nitrogen-containing carbon sphere (0.14 F/m^2^) [1], although the surface area of the HGCS was smaller.

The cycling stability was evaluated based on the capacitance retention after 2500 consecutive charge-discharge cycles between −1.0 to 0 V at a current density of 5 A/g (Figure 11e). The HGCS retained 94% of the initial capacitance after consecutive 2500 cycles, indicating their excellent electrochemical stability.

### 3.6. Adsorption Capacity of As-Prepared HGCS for MB

**Figure 12:**
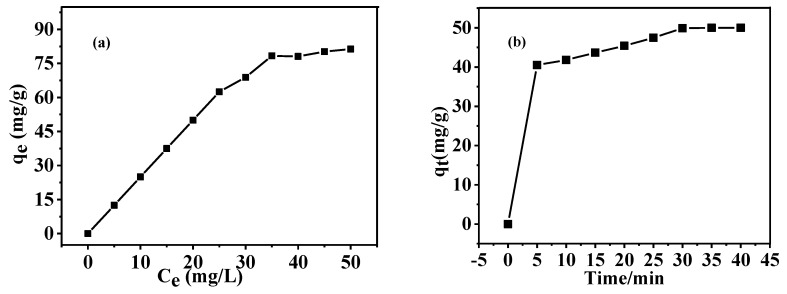
Adsorption isotherms (**a**) and adsorption kinetics (**b**) of HGCS prepared at 900 °C for 3 h with 1.0% Fe catalyst.

Adsorption behaviors of presynthesized carbon spheres and HGCS prepared at 900 °C/3 h with 1.0% Fe catalyst were also examined and compared using MB as an adsorbate. The adsorption isotherms (Figure S6 and Figure 12) of the samples were simulated using the Langmuir and Freundlich models expressed as follows:

(3) Langmuir isotherm: qe=Q0bce1+ce1n

(4) Freundlich isotherm: qe=kFce1n

where *q_e_* (mg/g) is the equilibrium adsorption amount, *Q*_0_ (mg/g) the maximum adsorption amount, *b* (L/mg) the constant term related to the energy of adsorption, *c_e_* (mg/L) the equilibrium concentration of dye solution, and *k_F_* and *n* are the Freundlich constants representing the adsorption capacity of the adsorbent and favorable extent of the adsorption process, respectively (n = 2–10, 1–2, and <1 indicates respectively good, moderate, and poor adsorption, respectively) [33].

The calculated adsorption parameters and correlation coefficients (R^2^) (Table 1) suggest that the Langmuir model fits better than the Freundlich model, indicating the dominant monolayer adsorption. Also, with the Freundlich model, n is between 1–2, indicating moderate adsorption. And the maximum monolayer adsorption capacity (*Q*_0_) of the as-prepared HGCS was calculated to be 182.8 mg/g, which is about 12 times as high as that (15.4 mg/g) in the case of the presynthesized carbon spheres. This can be mainly attributed to the much larger specific surface area and hierarchically porous structure of the former.

The following pseudo-first-order and pseudo-second-order kinetic models were also used to investigate the adsorption mechanism of the presynthesized carbon spheres and as-prepared HGCS (Figure S6d and Figure 12b);

(5) $$\mathrm{Pseudo}-\mathrm{first}-\mathrm{order}\;\mathrm{model}:\;q_{t}=q_{e}(1-e^{-k_{1}t})$$

(6) $$\mathrm{Pseudo}-\mathrm{second}-\mathrm{order}\;\mathrm{model}:\;\frac{t}{q_{t}}=\frac{1}{\kappa_{2}q_{e}^{2}}+\frac{t}{q_{e}}$$

where *q_t_* (mg/g) is the adsorption amount at time *t*, *k*_1_, and *k*_2_ (g·mg^–1^·min^–1^) are the pseudo-first-order and pseudo-second-order rate constants, respectively. Both the pseudo-first-order and the pseudo-second-order models describe the relationship between adsorption rate and MB concentration. As indicated in Table 1, the pseudo-second-order model shows a good linearity with correlation coefficients (R^2^) above 0.99, suggesting that the adsorption kinetics of the presynthesized carbon spheres and as-prepared HGCS both followed this model and the adsorption rate was irrelevant to the MB concentration.

## 4. Conclusions

Hierarchical micro-/mesoporous graphitic carbon spheres with an average size of 0.35 μm and specific surface area of 564 m^2^/g were synthesized by a facile combined hydrothermal carbonization and catalytic graphitization method using glucose as the carbon source and ferric nitrate as the catalyst precursor. The optimal weight ratio of Fe catalyst for graphitization of presynthesized amorphous carbon spheres was 1.0 wt %, and the optimal temperature and dwelling time were 900 °C and 3 h, respectively. The as-prepared HGCS exhibited a high specific capacitance of 140 F/g at 0.2 A/g, and good electrochemical stability with 94% capacitance retention after consecutive 2500 cycles. Moreover, the degree of graphitization contributed to 60% of the specific capacitance of the HGCS, this fundamental understanding provided an important clue for the development of high performance supercapacitor electrode materials. Moreover, the HGCS showed a good adsorption capacity of 182.8 mg/g for MB, which was 12 times as high as that of the presynthesized carbon spheres before graphitization.

## Figures and Tables

**Table 1:** Kinetics parameters of Langmuir and Freundlich models in the case of MB adsorption.

Samples	Langmuir			Freundlich			Pseudo-First-Order			Pseudo-Second-Order		
	Q_0_	B	R^2^	K_F_	N	R^2^	Q_e_	K_1_	R^2^	Q_e_	K_2_	R^2^
CS	15.4	0.024	0.95	0.38	1.21	0.95	47.4	0.336	0.969	5.05	0.283	0.999
HGCS	182.8	0.019	0.98	3.6	1.19	0.97	47.4	0.337	0.968	51.6	0.0125	0.995

## Supplementary Materials

The following are available online at http://www.mdpi.com/2079-4991/8/8/625/s1.
