# Supplementary material for: Preparation of Hierarchically Porous Graphitic Carbon Spheres and Their Applications in Supercapacitors and Dye Adsorption
Source: Nanomaterials (Basel). 2018 Aug 17;8(8):625. doi: 10.3390/nano8080625 (PMC6116252; doi:10.3390/nano8080625)
Supplement: Supplementary file 1 [file nanomaterials-08-00625-s001.pdf]

Supporting Information

Preparation of Hierarchically Porous Graphitic Carbon Spheres and Their Applications in Supercapacitors and Dye Adsorption

Saisai Li [1], Faliang Li [1], Junkai Wang [1], Liang Tian [1], Haijun Zhang [1,*] and Shaowei Zhang [2,*]

[1] The State Key Laboratory of Refractories and Metallurgy, Wuhan University of Science and Technology, Wuhan 430081, China; lisaisai281024@163.com (S.L.); lfliang@wust.edu.cn (F.L.); jkwang@hpu.edu.cn (J.W.); 13657244966@163.com (L.T.)

[2] College of Engineering, Mathematics and Physical Sciences, University of Exeter, Exeter EX4 4QF, UK

* Correspondence: zhanghaijun@wust.edu.cn (H.Z.); s.zhang@exeter.ac.uk (S.Z.);

Tel.: +86-27-68862829 (H.Z.); Fax: +86-27-68862829 (H.Z.)

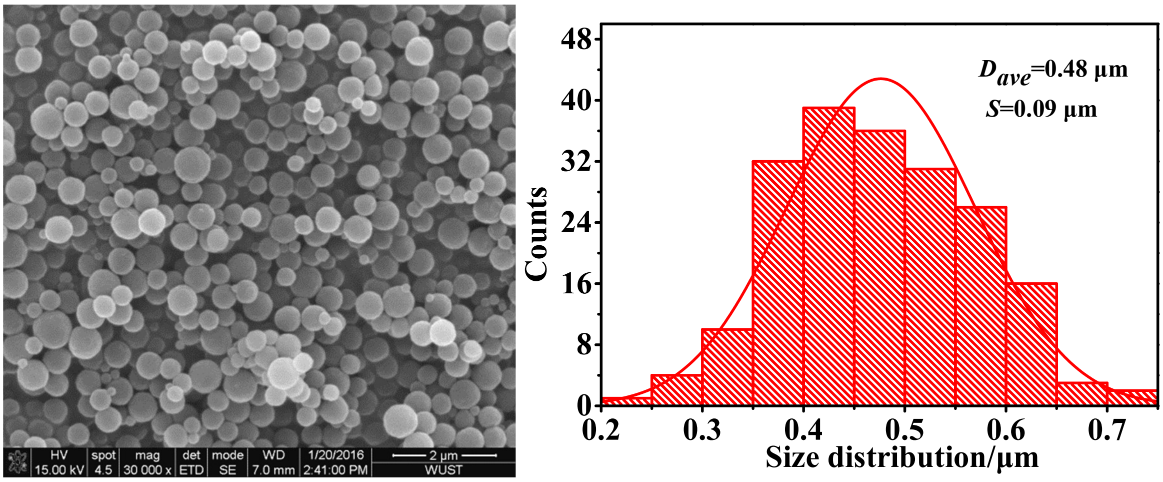

Fig. S1 SEM image and particle size distribution of carbon spheres prepared by hydrothermal carbonization at 180 °C for 12 h.

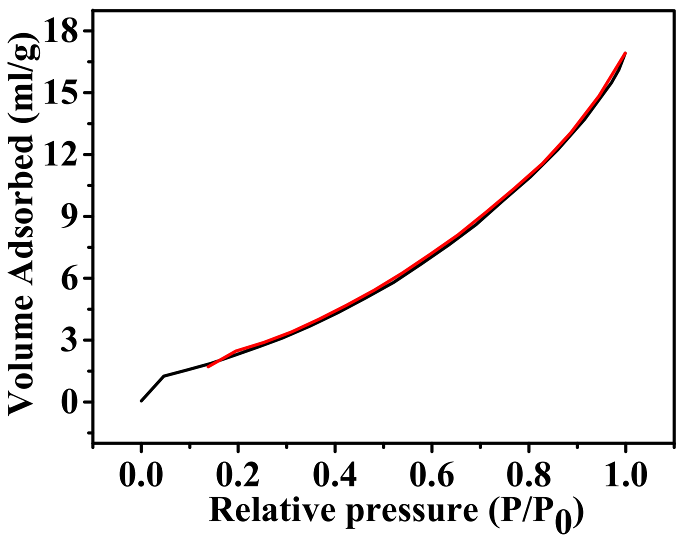

Fig. S2 Nitrogen adsorption/desorption isotherms of the pre-synthesized carbon spheres.

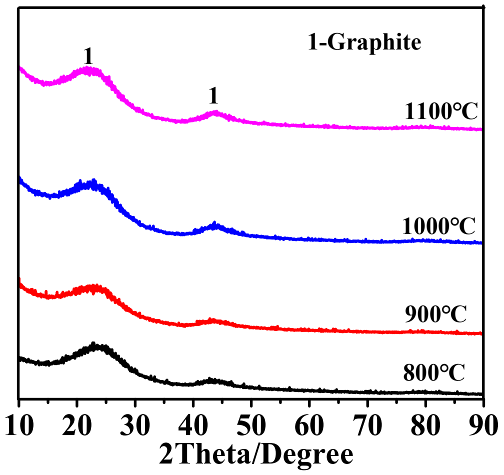

Fig. S3 XRD patterns of HGCS resultant from 3 h firing of pre-synthesized carbon spheres at different temperatures without using Fe catalyst (ICDD: 01-075-1621 (Graphite)).

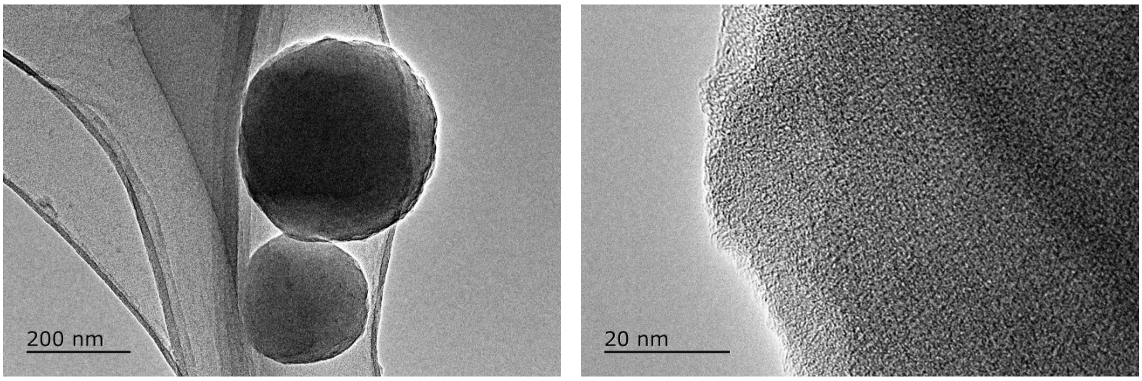

Fig. S4 TEM images of carbon spheres prepared by hydrothermal carbonization at 180 °C for 12 h.

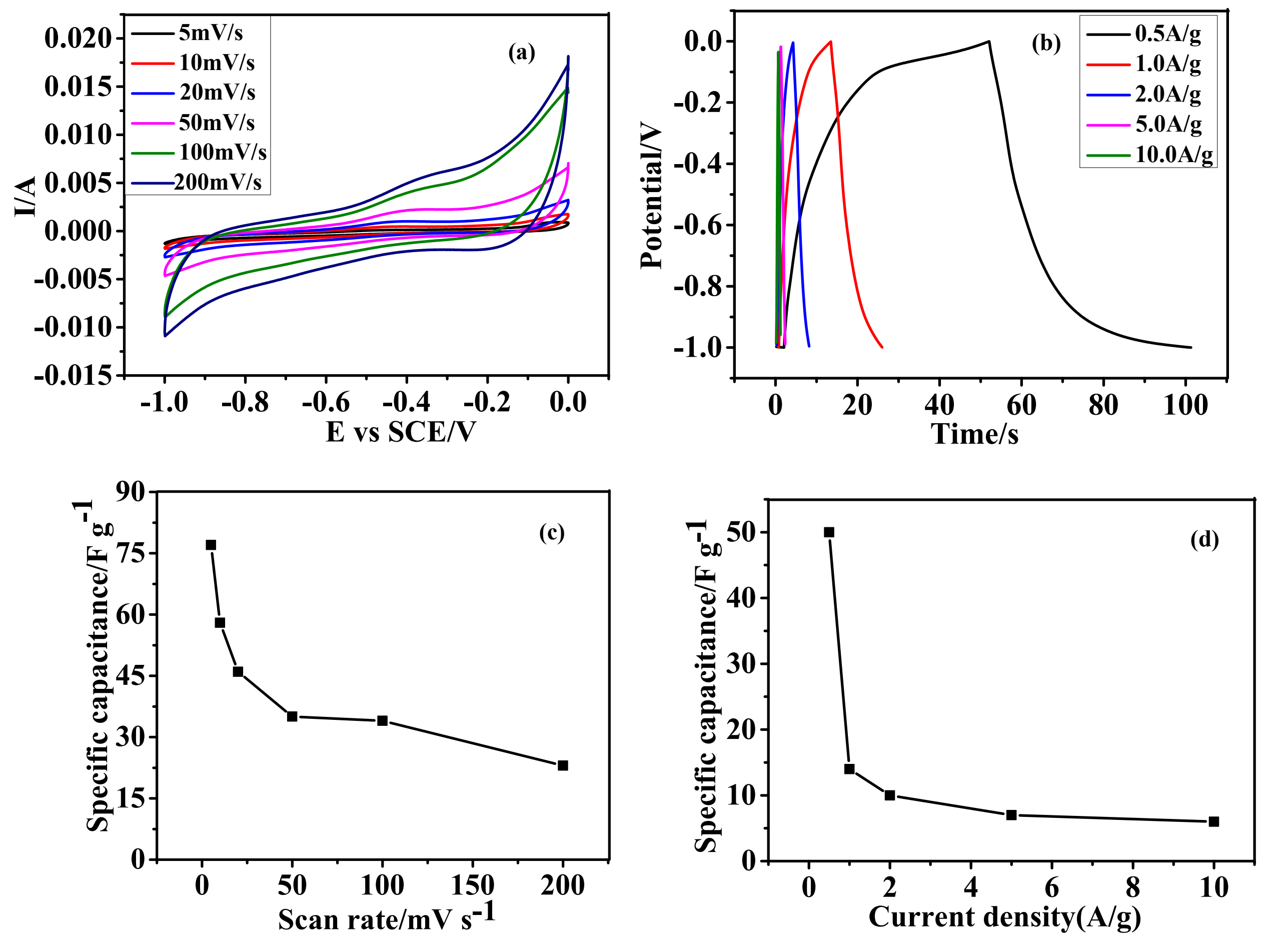

Fig. S5 CV curves (a), galvanostatic charge/discharge curves (b), specific capacitance versus scan rate (c) and specific capacitance versus current density (d), in the case of pre-synthesized carbon spheres.

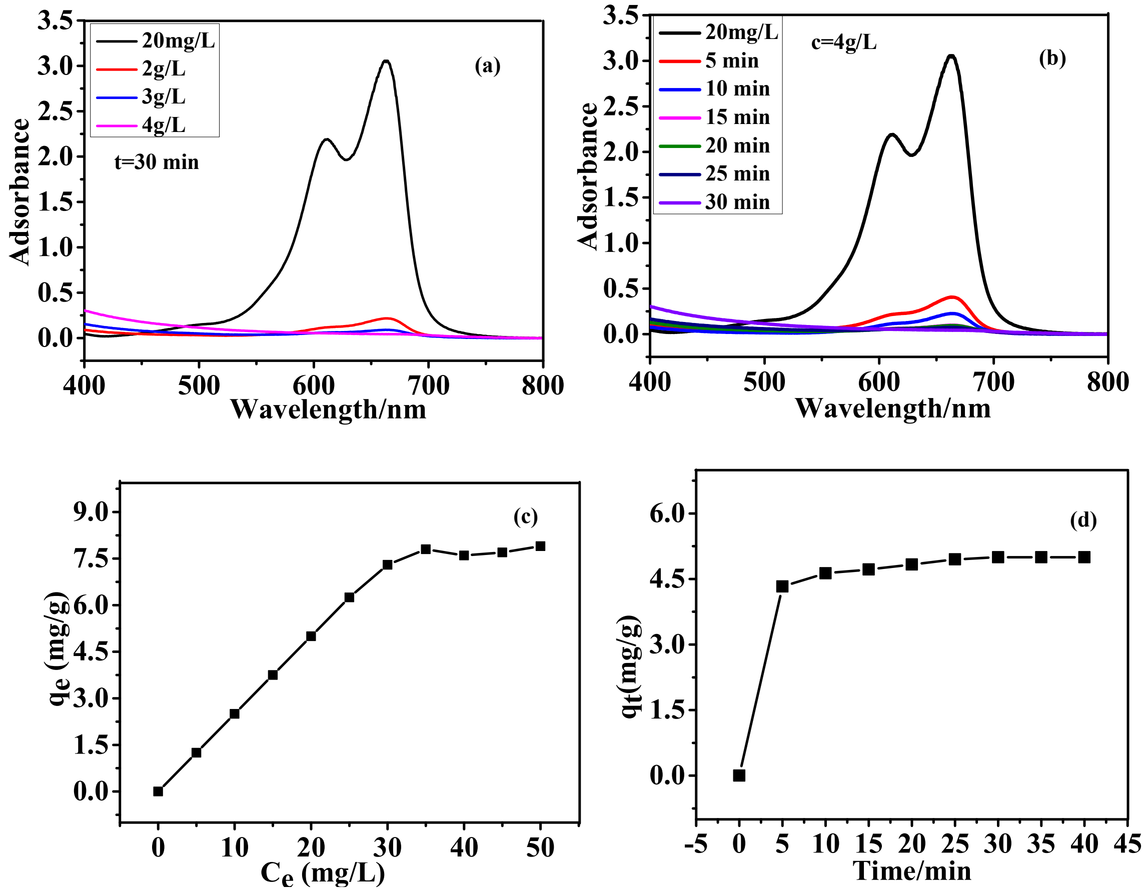

Fig. S6 UV curves corresponding to concentration (a) and time (b), adsorption isotherms (c), and adsorption kinetics (d), in the case of pre-synthesized carbon spheres.

Table S1 Comparison of graphitic carbon materials investigated to date

Samples	Carbon precursor	Preparation method	Specific surface area/m^2/g	Specific capacitance F/g	Refs
Mesoporous FeNi/graphitic carbon	Natural soybean oil	Template method	360~430	-	[1]
Nanoporous graphitic carbon	Iron phthalocyanine	Template method	960~1200	-	[2]
Porous graphitic carbon monoliths	Phenolic resol	Double template	725	-	[3]
Nanoporous graphitic carbon	Sucrose	Soft-template method	329	120 (0.2 A/g)	[4]
Mesoporous carbon spheres	Ethylene	Template method	666.8	59 (0.2 A/g)	[5]
HGCS	Glucose	Catalytic carbonization	564	140 (0.2 A/g)	This work

Table S2 Comparison of porous amorphous carbon materials reported to date

Samples	S_{BET} (m^2/g)	C (1A/g) (F/g)	$Cs=C/S_{BET}$ (F/m^2)	Refs
Hierarchical porous carbon nanospheres	1227	353	0.28	[6]
Activated hollow porous carbon spheres	1290	303.9	0.235	[7]
Nitrogen enriched carbon	1003	300	0.3	[8]
Hierarchically porous carbon spheres	1974	300	0.15	[9]
Hierarchical porous carbon	1513	300	0.198	[10]
Micro-meso-porous carbon spheres	2502	230	0.09	[11]

Hierarchical porous carbon spheres	1939	165	0.085	[12]
Activated carbon	1672	275	0.16	[13]
Nitro-doped ordered mesoporous carbons	1741	220	0.126	[14]
Hierarchically graphitic carbon spheres	564	113	0.2	This work

References

[1] Y. Wang, M. Yao, Y. Chen, Y. Zuo, X. Zhang, L. Cui, General synthesis of magnetic mesoporous FeNi/graphitic carbon nanocomposites and their application for dye adsorption, J. Alloy. Compd. 627 (2015) 7−12.

[2] P. Srinivasu, A. Islam, S. P. Singh, L. Han, M. L. Kantam, S. K. Bhargava, Highly efficient nanoporous graphitic carbon with tunable textural properties for dye-sensitized solar cells, J. Mater. Chem. 22 (2012) 20866−20869.

[3] Y. Liu, B. Lin, D. Li, X. Zhang, Y. Sun, H. Yang, Hierarchically porous graphitic carbon monoliths containing nickel nanoparticles as magnetically separable adsorbents for dyes, J. Appl. Polym. Sci. 132 (2015) 41322.

[4] M. Xie, J. Yang, J. Liang, X. Guo, W. Ding, In situ hydrothermal deposition as an efficient catalyst supporting method towards low-temperature graphitization of amorphous carbon, Carbon. 77 (2014) 215−225.

[5] K. Wilgosz, X. Chen, K. Kierzek, J. Machnikowski, R. J. Kalenczuk, E. Mijowska, Template method synthesis of mesoporous carbon spheres and its applications as supercapacitors, Nanoscale Res. Lett. 7 (2012) 1−5.

[6] S. W. L. Ng, G. Yilmaz, L. O. Wei, G. W. Ho, One-step activation towards spontaneous etching of hollow and hierarchical porous carbon nanospheres for enhanced pollutant adsorption and energy storage, Appl. Catal. B 220 (2017) 533−541.

[7] J. Liu, X. Wang, J. Gao, Y. Zhang, Q. Lu, M. Liu, Hollow porous carbon spheres with hierarchical nanoarchitecture for application of the high performance supercapacitors, Electrochim. Acta. 211 (2016) 183−192.

[8] X. Yang, C. Li, R. Fu, Nitrogen-enriched carbon with extremely high mesoporosity and

tunable mesopore size for high-performance supercapacitors, J. Power Sources. 319 (2016) 66−72.

[9] D. Zhang, J. Gao, Z. Li, S. He, J. Wang, Synthesis of hierarchically porous carbon spheres by an emulsification-crosslinking method and its application in supercapacitor, RSC Adv. 6 (2016) 54880−54888.

[10] Y. Han, S. Liu, D. Li, X. Li, Three-dimensionally hierarchical porous carbon creating high-performance electrochemical capacitors, Electrochim. Acta. 138 (2014) 193−199.

[11] Y. Fan, X. Yang, B. Zhu, P. F. Liu, H. T. Lu, Micro-mesoporous carbon spheres derived from carrageenan as electrode material for supercapacitors, J. Power Sources. 26 (2014) 584−590.

[12] J. Pang, W. Zhang, H. Zhang, J. Zhang, H. Zhang, G. Cao, Sustainable nitrogen-containing hierarchical porous carbon spheres derived from sodium lignosulfonate for high-performance supercapacitors, Carbon. 132 (2018) 280−293.

[13] L. Zhang, H. Gu, H. Sun, F. Cao, Y. Chen, G. Z. Chen, Molecular level one-step activation of agar to activated carbon for high performance supercapacitors, Carbon. 2018. doi.org/10.1016/j.carbon.2018.02.100

[14] Q. Shi, R. Zhang, Y. Lv, Y. Deng, A. A. Elzatahrya, D. Zhao, Nitrogen-doped ordered mesoporous carbons based on cyanamide as the dopant for supercapacitor, Carbon. 84 (2015) 335−346.
